# Thermodynamic Constants for Association of Isomeric Chlorobenzoic and Toluic Acids With 1,3-Diphenyl-guanidine in Benzene^1^

**DOI:** 10.6028/jres.065A.024

**Published:** 1961-06-01

**Authors:** Marion Maclean Davis, Hannah B. Hetzer

## Abstract

This paper reports values of Δ*F*_25_, Δ*H*, and Δ*S*_25_ for the association of diphenylguanidine with the isomeric monochlorobenzoic acids and the isomeric toluic acids in benzene from spectrophotometric measurements at 25 and 30 °C, using bromophthalein magenta E (3′, 5′, 3″, 5″-tetrabromophenolphthalein ethyl ester) as the indicator. The results are compared with available data for other donor-acceptor associations in aprotic solvents which include the monomer-dimer equilibrium of benzoic acids, the association of tertiary amines with iodine, and the association of certain oxygen bases with phenols. The comparisons indicate that the value of the ratio Δ*H*/298Δ*S* is approximately constant in the following associations in aprotic solvents: (1) Association of phenolic or carboxylic acids with nitrogenous bases to form hydrogen bonded ion-pairs; (2) hydrogen bonding of weakly acidic phenols to nitrogenous bases; (3) association of tertiary amines with iodine. A somewhat smaller value for this ratio seems to apply to most associations of phenols with oxygen bases. Possible applications of these findings include estimation of other thermodynamic constants when one of the constants Δ*F*, Δ*H*, or Δ*S* is known, and clarification of the relative importance of ionic and covalent contributions in hydrogen bond formation.

## 1. Introduction

A spectrophotometric procedure was described earlier [1],^2^ by means of which the relative strengths of 40 carboxylic acids of the benzoic acid series were determined when in benzene solution at 25 °C. The strengths were expressed as values of *K_assoc._* or log *K_assoc._* for the reaction

$$B\;\left(\text{base}\right)+\mathrm{HA}\;\left(\text{acid}\right)⇄{\mathrm{BH}}_{\ldots }^{+}A^{-},$$ (1)

in which the base used was 1,3-diphenylguanidine. The phenolic acid, bromophthalein magenta E (3′,5′,3″, 5″- tetrabroinophenolphthalein ethyl ester) served as the indicator dye. An essential step was determining *K* for the association of diphenylguanidine with bromophthalein magenta E. After completion of the experimental work at 25 °C [1], analogous measurements at 30 °C were made for part of these acids, namely, bromophthalein magenta E, benzoic acid, the isomeric chlorobenzoic acids, and the isomeric toluic acids. This paper reports thermodynamic constants derived from the combined data at 25 and 30 °C, and discusses their significance.

## 2. Experimental Procedure and Results

Among the possible sources of error in studies of acid-base equilibria in benzene by spectrophotometry are the volatility of the solvent and effects of adventitious moisture or oxygen. Such errors can be minimized by making absorbance measurements very soon after the preparation of solutions.^3^ To facilitate speed in obtaining optical data, as well as to reduce errors arising from imprecise temperature control, the temperature in our laboratory is automatically controlled to match closely the temperature within the thermostated air bath which serves as the absorption cell compartment [3]. Throughout most of the year, a laboratory temperature of 25±0.5 °C can be maintained. During summer and winter weather, respectively, the laboratory temperature can be held as high as 30 °C or as low as 20 °C.

Qualitative observations of thermal effects on the extent of association of bromophthalein magenta E with bases [4] had made it seem likely that enthalpy and entropy changes involved in the kinds of acid-base associations which have been under study could be estimated from optical measurements covering the temperature range 20 to 30 °C, and plans were made to extend the measurements already made at 25 °C [1] to these two additional temperatures. In August and September of 1955 some of the experiments (see introduction) were repeated at 30 °C, using the same materials and following the same experimental technique as at 25 °C, but the work had to be interrupted without performance of the intended measurements at 20 °C.

The combined results of the experiments at 25 and 30 °C are summarized in table 1.^4^ The steps followed in calculating association constants corresponding to eq (1), and then applying a correction for the amount of carboxylic acid dimer presumed to be present, were the same as previously explained [1]. As noted in table 1, parallel studies of the association of diphenylguanidine with bromophthalein magenta E and with benzoic acid at 25 and 30 °C have been made in this laboratory [5], with very similar results.

The dimer-monomer data used in making the corrections are not known with certainty to be accurate. Self-association of diphenylguanidine, formation of complex anions (RCOOHOCOR)^−^, the “secondary” reaction of diphenylguanidine with bromophthalein magenta E [6], and adsorption of solutes on glass-or silicaware are additional possible causes of errors. However, it is believed that experimental uncertainties in the optical data are the main obstacle to the attainment of high accuracy; these have more effect when the association constants are relatively great in magnitude (10^5^ to 10^6^), as in the present work.^5^

Values for the thermodynamic constants Δ*F*_25_, Δ*H*, and Δ*S*_25_ were calculated in the conventional way from the *K*_assoc._ values at the two temperatures. These results are summarized in table 2.^6^

## 3. Discussion

Two well-known thermodynamic equations,

−RTlnK=ΔF (2)

and

ΔF=ΔH−TΔS, (3)

are frequently utilized in efforts to develop generalizations about the effects of structural modifications on reaction rates and equilibria (for example, see [7 to 13]). The great majority of the attempts to assess relative contributions of changes in enthalpy and entropy to free energy changes have been made in connection with studies of reaction kinetics. In some instances, a structural change apparently leads to an increase in the energy of activation, with little or no effect on the entropy factor.^7^ In other cases, an increase in the energy of activation is accompanied by a parallel effect on the entropy factor.^8^

A class of chemical equilibria which is of major interest is the ionization of acids of the benzoic acid series in water, since this reaction series was adopted for evaluating substituent constants in the Hammett equation [8]. Thermodynamic constants for the aqueous ionization of some benzoic acids pertinent to this paper are compiled in table 3. The Δ*H* values for these acids, except in the case of *o*-toluic acid, are much less than one kcal mole^−1^ in magnitude. Clearly, the values of Δ*F*_25_ for aqueous ionization of the acids depend almost solely on the temperature-entropy term, *T*Δ*S*.

The thermodynamic constants obtained in this work for association of benzoic acids with 1,3-diphenylguanidine in benzene (table 2) contrast greatly with the corresponding constants for aqueous ionization.^9^ Values of Δ*H* are in the approximate range 16 to 19 kcal mole^−1^, are all negative in sign, and the variations in numerical magnitude parallel those in the temperature-entropy term, the ratio Δ*H*/298Δ*S*_25_ being 1.8, or very close to this value, in all cases. For five of the reactions Δ*H/*Δ*F*_25_ is 2.3 the extreme values being 2.1 and 2.4.

**Figure 1:**
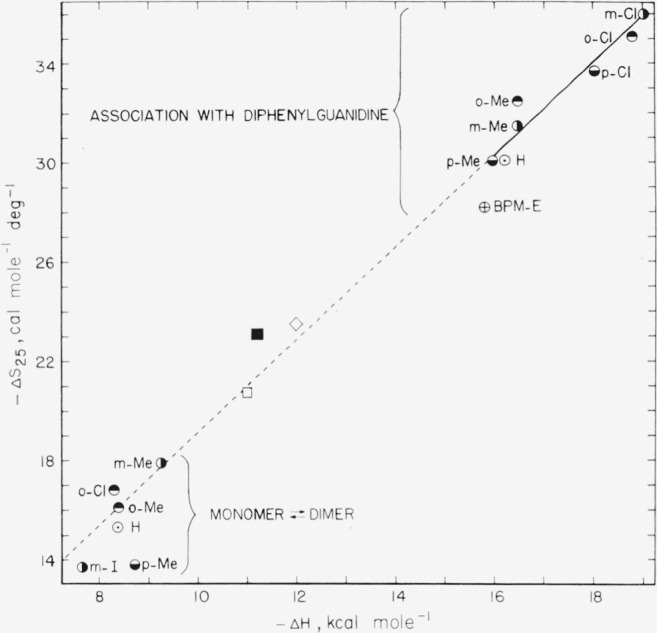
−ΔS_25_ versus −ΔH for association of some benzoic acids with 1,3-diphenylguanidine and for self-association (dimerization) of the same or analogous acids, in benzene. H, Benzoic acid; *o*-, *m*-, and *p*-Me, the toluic acids; *o*-, *m*-, and *p*-Cl,themono-chlorobenzoic acids; BPM-E, bromophthalein magenta E; see table 2. The unlabeled symbols refer to the following associations (see table 5): open square, triethylamine with benzoic acid in benzene; filled square, tribenzylamine with picric acid in benzene; open diamond, triethylamine plus iodine in *n*-heptane.

For comparison, available values of Δ*H* and Δ*S*_25_ for the monomer-dimer equilibrium of the same or closely related benzoic acids are presented in table 4. It is of considerable interest that the ratio Δ*H*/298Δ*S*_25_ has practically the same value for the self-association of the benzoic acids in benzene as for their association with diphenylguanidine in this solvent.^10^ The enthalpy-entropy relationship for the two kinds of association reactions is also brought out in figure 1, in which −Δ*S*_25_ is plotted against −Δ*H.* In this figure the equation for the *solid* line was calculated by the method of least squares, using data for all seven of the carboxylic acids.^11^ The *dashed* line is an extension of the solid line. For the monomer-dimer equilibrium, Δ*H/*Δ*F*_25_ varies from 2.1 to 2.5, with the exception of *p*-toluic acid where the value is 1.9.^12^

A few additional investigations of thermodynamic properties of acid-base associations in aprotic solvents have been reported. Table 5 is a compilation of most of the published data. Several tentative conclusions may be drawn:

1. In most associations in which nitrogen is the proton acceptor (electron donor), the value of Δ*H*/298Δ*S*_25_ is not far from 1.8.^13^
2. Statement (1) holds irrespective of whether the acid is a Brønsted acid (phenol, carboxylic acid) or the Lewis acid iodine.
3. In most associations in which oxygen is the proton acceptor (electron donor), the value of Δ*H*/298Δ*S*_25_ is 1.4.^14^ Results with dioxane and methyl or ethyl acetate as the base indicate that the values of both −Δ*H* and −Δ*S*_25_ increase on changing from carbon tetrachloride to a saturated hydrocarbon solvent, but without affecting the ratio of the two values.
4. With benzene as base (*π*-electron donor), the value of Δ*H*/298Δ*S* seems to be smaller.^15^

A linear relationship of −Δ*H* and −Δ*S* in a related series of association reactions has been interpreted as signifying that with increasing strength of the bond between donor and acceptor there is increased restraint on motions of the component parts.^16^ Perhaps the different values which Δ*H*/298Δ*S*_25_ seems to have (see table 5) for bases with nitrogen as the electron donor atom and those with oxygen as the electron donor (approximately 1.8 and 1.4, respectively) result chiefly from variations in −Δ*H.*

The existent thermodynamic data for donor-acceptor reactions in aprotic solvents are not extensive enough to determine the scope of the relationships indicated above, or accurate enough to detect possible small effects resulting from variations in structure as, for example, isomerism in the toluic acids or the chlorobenzoic acids. Two important areas of possible application may be suggested, however.

(1) If one of the three constants Δ*F*, Δ*H*, and Δ*S* is known, it should be possible to estimate values for the other two. To illustrate, from the value of Δ*H* which has been determined for association of pyridine with methanesulfonic acid in nitrobenzene (see table 5), estimated values for Δ*S*_25_, Δ*F*, and *K*_assoc._, in the units used above, are −32, −7.6, and 3.7×10^5^, respectively. The assumption must be made that nitrobenzene does not affect the association differently from other solvents listed in the table. A further example concerns association of triethylamine with phenol in *n*-heptane. The reported value of *K*_assoc._ at 25 °C is 83.8 [21]. This leads to the following approximate values for Δ*F*, Δ*H*, and Δ*S*_25_, respectively: −2.6, −5.9, and −11. These agree well with the constants reported for association of trimethylamine with phenol in cyclohexane (see table 5).

(2) The theory of hydrogen bond formation still needs clarification (see [17], chs. 7 and 8). Evidence for hydrogen bonding in ion-pairs of salts that are formed by union of nitrogenous bases with hydrogen acids (and therefore have one or more protons attached to the nitrogen of the cation) has been pointed out ([4,2,5] and references cited), but has not received wide consideration in discussions of hydrogen bonding. The thermodynamic data in tables 2, 4, and 5 point to a relationship of such systems with the more weakly bonded systems which so far have been the basis for speculations about the nature of hydrogen bonding and the relative importance of ionic and covalent contributions.

## Figures and Tables

**Table 1:** Equilibrium constants for association of acids with 1,3-diphenylguanidine in benzene at 25 and 30 °C

Acid	t (°C)	No. expts.	Range of *n*^a^	Range of *n″*^a^	10^3^*K*_21_^b^	10^−5^*K*_cor._^b^	St. dev.	Coeff. var. %
								
Bromophthalein magenta E	$\{\begin{array}{c} 25\\ 30 \end{array}$	10	0.25 to 3	………………………………	………………………………	2.55 2.57^c^	0.098	3.8
		8	.75 to 3	………………………………	………………………………	1.649 1.66^c^	.070	4.3
Benzoic	$\{\begin{array}{c} 25\\ 30 \end{array}$	25	1 to 3	0.5 to 5	1.6	2.01 2.09^c^	.045	2.2
		8	0.5 to 3	.5 to 3	2.0	1.28 1.31^c^	.081	6.3
*o*-Chlorobenzoic	$\left\{ \begin{array}{l} 25\\ 30 \end{array}\right.$	9	.5 to 3	.5 to 4	3.7	12.5	.047	3.8
		8	.5 to 3	.5 to 4	4.7	7.42	.235	3.2
*m*-Chlorobenzoic	$\left\{ \begin{array}{l} 25\\ 30 \end{array}\right.$	16	.5 to 3	.5 to 5	2.4	12.1	.023	1.9
		8	.5 to 4	.5 to 4	3.0	7.13	.116	1.6
*p*-Chlorobenzoic	$\left\{ \begin{array}{l} 25\\ 30 \end{array}\right.$	8	.5 to 3	.5 to 4	(1.7)	6.94	.133	1.9
		9	.5 to 3	.5 to 4	(2.0)	4.20	.108	2.6
*o*-Toluic	$\left\{ \begin{array}{l} 25\\ 30 \end{array}\right.$	19	.5 to 3	.5 to 8	2.4	0.930	.179	1.9
		8	1 to 3	.25 to 4	3.0	.588	.112	1.9
*m*-Toluic	$\left\{ \begin{array}{l} 25\\ 30 \end{array}\right.$	11	1 to 3	1 to 8	1.4	1.50	.041	2.7
		9	0.5 to 3	0.5 to 4	1.8	0.948	.219	2.3
*p*-Toluic	$\left\{ \begin{array}{l} 25\\ 30 \end{array}\right.$	10	1 to 3	1 to 4	0.415	1.34	.084	6.3
		6	0.5 to 3	0.5 to 3	0.53	0.859	.288	3.4

a The molar concentration (C_a_) of bromophthalein magenta E (3′, 5′, 3″, 5″-tetrabromophenolphthalein ethyl ester) was 5.0 × 10^−5^ throughout. The molar concentrations of 1,3-diphenylguanidine (*n*C_a_) and of the aromatic carboxylic acid (*n*″*C*_a_) varied within the ranges indicated. Experimental procedure and apparatus were as described in [1].

b *K*_21_ is the equilibrium constant for dissociation of dimeric carboxylic acid into the monomer in benzene, while *K*_cor._ is the equilibrium constant for the association A (acid) +B (base)⇆S(salt) in benzene after the raw data have been corrected by taking into consideration the dimer-monomer equilibrium of the carboxylic acid; for method of calculating *K*_cor_. see [1], especially sections 3.2 and 4.1. The values of *K*_21_ used in correcting the association constants were based on data in the literature except in the cases of *m*- and *p*-chlorobenzoic acids, for which *K*_21_ values have not been reported. In the case of *m*-chlorobenzoic acid, data for *m*-iodobenzoic acid were applied; in that of *p*-chlorobenzoic acid, *K*_21_ values at 25 and 30 °C were estimated by trial and error. All equilibrium constants given in this table are in molar units.

c Reference [5].

**Table 2:** Thermodynamic constants for association of acids with 1,3-diphenylguanidine in benzene^a^

Acid	Δ*F*_25_	Δ*H*	Δ*S*_25_	$\frac{\Delta H}{298\Delta S_{25}}$
				
	*kcal* *mole*^−1^	*kcal* *mole*^−1^	*cal* *mole*^−1^ *deg*^−1^	
Bromophthalein magenta E	−7.38	15.8	−28.2	1.9
Benzoic	−7.24	−16.2	−30.1	1.8
*o*-Chlorobenzoic	−8.32	−18.8	−35.1	1.8
*m*-Chlorobenzoic	−8.30	−19.0	−36.0	1.8
*p*-Chlorobenzoic	−7.97	−18.0	−33.7	1.8
*o*-Toluic	−6.78	−16.5	−32.5	1.7
*m*-Toluic	−7.06	−16.5	−31.5	1.8
*p*-Toluic	−7.00	−16.0	−30.1	1.8

a Calculated from association constants expressed in liter mole^−1^ units. See discussion in section 2 of the text.

**Table 3:** Thermodynamic constants for ionic dissociation of selected benzoic acids in water

Acid	ΔF_25_	ΔH	ΔS_25_	298ΔS_25_
				
	*kcal* *mole*^−1^	*kcal mole*^−1^	*cal mole*^−1^ *deg*^−1^	*kcal* *mole*^−1^
Benzoic	5.74	0.11^a^ 0.104^c^ 0.09^d^	−18.9^a^ ^c^ ^d^	−5.64
*m*-Chlorobenzoic	5.22	.019^b^	−17.4^b^	−5.19
*m*-Iodobenzoic	5.26	.190^b^	−17.0^b^	−5.07
*p*-Chlorobenzoic	5.43	.226^b^	−17.5^b^	−5.22
*o*-Toluic	5.33	−1.50^d^	−22.9^d^	−6.83
*m*-Toluic	5.78	0.07^b^ ^d^	−19.2^b^ ^d^	−5.72
*p*-Toluic	5.92	0.30^b^ 0.24^d^	−19.0^d^	−5.67

a T. L. Cottrell, G. W. Drake, D. L. Levi, K. J. Tully, and J. H. Wolfenden, J. Chem. Soc. (London) **1948**, 1016.

b G. Briegleb and A. Bieber, Z. Elektrochem. **55**, 250 (1951).

c A. V. Jones and H. N. Parton, Trans. Faraday Soc. **48**,8 (1952).

d T. W. Zawidski, H. M. Papée, and K. J. Laidler, Trans. Faraday Soc. **55**, 1743 (1959).

**Table 4:** Thermodynamic constants for self-association of aromatic acids in benzene^a^

Acid	Δ*F*_25_	Δ*H*^d^	Δ*S*_25_^d^	$\frac{\Delta H}{298\Delta S_{25}}$^d^
				
	*kcal* *mole*^−1^	*kcal* *mole*^−1^	*cal* *mole*^−1^ *deg*^−1^	
Benzoic	−3.81	−8.37	−15.3	1.8
*o*-Chlorobenzoic^b^	−3.31	−8.31	−16.8	1.7
*m*-Iodobenzoic^b^	−3.57	−7.65	−13.7	1.9
*o*-Toluic^c^	−3.58	−8.39	−16.1	1.7
*m*-Toluic^c^	−3.91	−9.26	−17.9	1.7
*p*-Toluic^b^	−4.61	−8.72	−13.8	2.1

a Calculated from monomer-dimer equilibrium constants (*K*_12_) expressed in liter mole^−1^ units.

b Computed from data of G. Allen and E. F. Caldin (ref. [18]), after converting monomer-dimer constants from mole fraction units to liter mole^−1^ units.

c From data of F. T. Wall and F. W. Banes, J. Am. Chem. Soc. **67**, 898 (1945).

d Dividing these values of Δ*H* and Δ*S*_25_ by two, so as to obtain the average values per hydrogen bond has no effect, of course, on the ratio Δ*H/T*Δ*S.*

**Table 5:** Thermodynamic constants for miscellaneous acid-base associations in aprotic solvents^a^

Solvent	Base	Acid	Δ*F*_25_	Δ*H*	Δ*S*_25_	$\frac{\Delta H}{298\Delta S_{25}}$
						
			*kcal* *mole*^−1^	*kcal* *mole*^−1^	*cal* *mole*^−1^ *deg*^−1^	
Benzene^b^	Triethylamine	Bromophthalein magenta E	−6.1	−15.3	−30.9	1.7
Benzene^b^	Triethylamine	Benzoic acid	−4.9	−11.0	−20.7	1.8
Benzene^c^	Tribenzylamine	Picric acid	−4.4	−11.2	−23.1	1.6
Carbon tetrachloride^d^	Benzene	Iodine	+1.1	−1.1	−7.4	0.5
Carbon tetrachloride^e^	Dioxane	Phenol	………………	−4.7	−11.5	1.4
Carbon tetrachloride^f^	Diethyl ether	Phenol	………………	−3.7	−7.6	1.6
Carbon tetrachloride^g^	Ethyl acetate	Phenol	………………	−4.8	−11.9	1.4
Carbon tetrachloride^f^	Hexamethylenetetramine	Phenol	………………	−6.9	−13.6	1.7
Chlorobenzene^h^	*n*-Butylamine	2,4-Dinitrophenol	−3.1	−12.2	−30.5	1.3
Chlorobenzene^h^	Di-*n*-butylamine	2,4-Dinitrophenol	−4.6	−11.4	−22.8	1.7
Chlorobenzene^h^	Tri-*n*-butylamine	2,4-Dinitrophenol	−5.1	−14.4	−31.3	1.5
Cyclohexane^i^	Trimethylamine	Phenol	−2.6	−5.8	−10	1.9
Cyclohexane^i^	Trimethylamine	*p*-Chlorophenol	−3.1	−7.0	−13	1.8
Cyclohexane^i^	Trimethylamine	*p*-Cresol	−2.4	−3.8	−5	2.6
*n*-Heptane^j^ ^o^	Pyridine	Iodine	−3.3^j^	−7.8	−15.5^j^	1.7^j^
*n*-Heptane^k^ ^o^	Triethylamine	Iodine	−5.0	−12.0	−23.5	1.7
*n*-Heptane^l^	Methyl acetate	Phenol	………………	−5.3	−12.8	1.4
Isooctane^m^	*N,N*- Dimethylacetamide	Phenol	………………	−7.7	−14.8	1.8
Petroleumether^e^	Dioxane	Phenol	………………	−5.4	−13.1	1.4
Petroleumether^g^	Ethyl acetate	Phenol	………………	−5.7	−13.7	1.4
Nitrobenzene^n^	Pyridine	Methanesulfonicacid	………………	−17.1	………………	………………

a From thermodynamic constants or association constants (converted where necessary to liter mole^−1^ units) given in the references cited below.

b Reference [5].

c A. A. Maryott, J. Research NBS **41**, 7 (1948); M. M. Davis and E. A. McDonald, J. Research NBS **42**, 595 (1949).

d Bee reference [5], table II, footnote *f*.

e S. Nagakura, J. Chem. Soc. Japan, Pure Chem. Sect. **74**, 153 (1953), through reference [17], appendix B.

f M. Tsuboi, Bull. Chem. Soc. Japan **25**, 60 (1952).

g S. Nagakura, J. Am. Chem. Soc. **76**, 3070 (1954).

h See reference [5], table II, footnote *d.*

i R. L. Denyer, A. Gilchrist, J. A. Pegg, J. Smith, T. E. Tomlinson, and L. E. Sutton, J. Chem. Soc. (London) **1955**, 3889; see table 5.

j C. Reid and R. S. Mulliken, J. Am. Chem. Soc. **76**, 3869 (1954). Δ*F* and Δ*S* are for 17 °C instead of 25 °C.

k S. Nagakura, J. Am. Chem. Soc. **80**, 520 (1958).

l S. Nagakura, J. Chem. Soc. Japan, Pure Chem. Sect. **75**, 734 (1954), through reference [17], appendix B.

m S. Mizushima, M. Tsuboi, T. Shimanouchi, and Y. Tsuda, Spectrochim. Acta **7**, 100 (1955).

n H. C. Brown and R. R. Holmes, J. Am. Chem. Soc. **77**, 1727 (1955).

o The method used in calculating *K*_assoc._ values has been criticized (see R. S. Drago and N. J. Rose, J. Am. Chem. Soc. **81**, 6141 (1959).

## Footnotes

2 Figures in brackets indicate the literature references at the end of this paper.

3 For example, leakage of atmospheric oxygen into glass-stoppered absorption cells containing the base tribenzylamine in benzene solution was found to cause its partial conversion to tribenzylamine oxide. To determine the equilibrium constant for association of tribenzylamine with bromophthalein magenta E, the benzene was freshly boiled to remove dissolved air and the measurements were made as quickly as possible [2].

4 The raw data reported for 25 °C [1] are typical of those obtained at 30 °C.

5 See [5], footnote 10.

6 The values of Δ*H* and Δ*S*_25_ given in table II of [5] for the association of diphenylguanidine with bromophthalein magenta E and benzoic acid were based on *K*_assoc_. values at 20, 25, and 30 °C. Unaccountably the value of *K*_assoc_. found at 20 °C for the reaction of diphenylguanidine with bromophthalein magenta was lower than the value expected (~4×10^5^) and, as a result, the value of *K*_assoc_. for the reaction of diphenylguanidine with benzoic acid was also lower than expected. If 4×10^5^ is assumed to be approximately the correct *K* value for the association of diphenylguanidine with bromophthalein magenta at 20 °C, *K* for its association with benzoic acid acquires the expected magnitude, and the thermodynamic constants obtained agree closely with the ones given in table 2 of this paper.

7 For example, in the *alkaline* hydrolysis of *m*- and *p*-substituted ethyl benzoates in 85-percent aqueous ethanol [14].

8 An illustration is the *acidic* hydrolysis of *m*- and *p*-substituted ethyl benzoates in 85-percent aqueous ethanol [15].

9 In comparing relative acidic strengths in water and benzene, one must bear in mind that the primary acid-base reaction eq (1) is a shift of the acid proton toward the base to form hydrogen-bonded ion-pairs. Formation of independently moving ions occurs to a very minor extent unless the ions can become hydrogen-bonded to molecules of solvent or solute [3, 6]. This “secondary reaction” is likely to hamper study of the main acid-base interaction.

10 Others have recognized a linear correlation of Δ*H* and Δ*S* in the dimerization of carboxylic acids, as well as in some additional instances of hydrogen bond formation [16, 17], but the units of concentration were not the same as those used here, thus leading to different values for Δ*H* and Δ*S.*

11 Omitting data for *o*-toluic acid would have given a still better correlation of Δ*H* with Λ*S*_25_ values.

12 *p*-Toluic acid shows anomalous behavior in the comparison of *K*_12_ values for carboxylic acids in benzene with the *K*_ion_. values in water [16]. Its tendency to dimerize in benzene [18] seems surprisingly great.

13 The thermodynamic constants for the three base-acid systems in cyclohexane are small, and the authors regarded the values as being rough. Nevertheless, the agreement of two of the values of ΔH/298Δ*S*_25_ with those for stronger acid-base interactions is very good.

14 The monomer-dimer equilibrium of benzoic acids (see table 4) does not conform to this generalization.

15 The very interesting thermodynamic data for formation of alkylbenzeneiodine [19] and alkylbenzene-iodine monochloride [20] complexes in carbon tetrachloride solution, which show a linear correlation of Δ*H* and Δ*S*, are in other units of concentration, and raw data necessary for conversion to molar units were not published. Consistent use of molar units of association in presenting thermodynamic properties of such association reactions would have the advantage of facilitating comparisons with systems containing both donor and acceptor compounds in concentrations much too low for use of mole fraction units.

16 For example, see [17, 19, 20].
